# Expression Pattern of Neuronal Markers in PB-MSCs Treated by Growth Factors Noggin, bFGF and EGF

**Published:** 2015

**Authors:** Zahra Fazeli, Sayyed Mohammad Hossein Ghaderian, Masoumeh Rajabibazl, Siamak Salami, Nader Vazifeh Shiran, Mir Davood Omrani

**Affiliations:** 1 *Department of Medical Genetics, Faculty of Medicine, Shahid Beheshti University of Medical Sciences, Tehran, Iran.*; 2 *Urogenital Stem Cell Research Center, Shahid Beheshti University of Medical Sciences, Shahid Labbafi Nejad Educational Hospital, Tehran, Iran.*; 3 *Department of Clinical Biochemistry, Faculty of Medicine, Shahid Beheshti University of Medical Sciences, Tehran, Iran.*; 4 *Department of Hematology, School of Allied Medical Sciences, Shahid Beheshti University of Medical Sciences, Tehran, Iran.*

**Keywords:** Mesenchymal stem cells, differentiation, neuronal markers, Noggin

## Abstract

Mesenchymal stem cells (MSCs) have the ability to differentiate into neuronal like cells under appropriate culture condition. In this study, we investigated whether MSCs derived from human peripheral blood (PB-MSCs) can differentiate into neuronal like cells by synergic effect of the growth factors EGF, bFGF and Noggin. For this purpose, the expression of five neuronal markers (Nestin, β III tubulin, *NFM*, *MAP2* and *NSE*) were evaluated in treated PB-MSCs by SYBR Green Real time PCR. The expression analysis showed a higher expression of β-tubulin and *NFM* in treated BP-MSCs compared with untreated PB-MSCs as a control group. The expression of Nestin was also diminished in PB-MSCs treated with Noggin. This study suggested that the treatment of PB- MSCs with Noggin alongside with bFGF and EGF might differentiate these cells into neuronal lineage cells. The obtained results could be further developed for useful applications in regenerative medicine.

Nowadays, the regenerative medicine has provided an alternative source of different cell lines and organs through trans- differentiation process (1). During last decades, different strategies have been used to regenerate missing tissues (2). Some studies indicated that stem cells derived from fetal umbilical cord have the ability to differentiate into cell types of different organs (3-4). In other alternative methods, the induced pluripotent stem cells (iPSCs) generated from somatic cells have opened a new window in regenerative medicine (5). However, the use of these cells was accompanied by some limitations. For example, lentivirus vectors including transcription factors for transformation of somatic cells into iPSCs could cause the recipient cells to exhibit long-term genetic aberrations (6). The characterization and isolation of stem cells from different organs and tissues has represented an alternative source of cells in cell therapy or regenerative medicine. However, it is not possible to isolate stem cells from some tissues including central nervous system (CNS). Therefore, trans-differentiation of stem cells derived from other tissues could provide a suitable supply for regeneration of these tissues. Current studies have demonstrated that the mesenchymal stem cells (MSCs) derived from different tissues have the ability to differentiate into different cell types.

MSCs are multipotent cells that can differentiate into chondrocytes, osteocytes, adipocytes, myocytes, endothelial cells, and neurons (7-10). Although the primary sources of MSCs are bone marrow, umbilical-cord blood, olfactory bulb, amniotic fluid (AF), and Wharton’s jelly, these cells have also been found in peripheral blood (11-12). Studies have demonstrated that MSCs have the ability to spontaneously express the neural markers including nestin, NeuN, gilal fibrillary acidic protein (GFAP) and βIII tubulin (13). These observations could support the predisposition of MSCs to differentiate toward neuronal lineage cells including neurons, oligodendrocytes and astrocytes. Different protocols have been published for differentiation of MSCs into neuronal lineage cells (9, 14-16). There is increasing evidence about neural induction of MSCs by numerous growth factors (9, 17). These growth factors are able to regulate neuronal differentiation through different mechanisms (18). It has been known that BMP2 is one of the most important *bone* morphogenetic proteins (BMPs) in regulating the osteogenic differentiation (19). Previous studies indicated that the inhibition of BMP2 by Noggin prevented from osteogenic differentiation through blockage of Smad signalling (20-21). Furthermore, it has been demonstrated that the inhibition of BMP signalling by Noggin along with activation of bFGF signalling could participate into neural differentiation of MSCs (22).

In this study, we attempted to differentiate peripheral blood derived MSCs (PB-MSCs) into neuronal cells by inhibition of BMP signalling upon treatment with growth factors such as Noggin, bFGF and EGF. The expression of neural markers like nestin, β *III *tubulin, *neurofilament* M (NFM), *microtubule-* *associated protein 2 (*MAP2) and *neuron-specific enolase* (NSE) in treated cells were investigated to determine whether those growth factors could influence the expression of these neural markers.

## Materials and methods

**PB-MSCs isolation**

The peripheral blood (almost 6 ml) was obtained from three healthy individuals. The blood was collected in EDTA-treated tubes and layered over ficoll in a 2:1 ratio. The peripheral blood mononuclear cells (PBMCs) were separated by density gradient centrifugation, plated in DMEM/F-12 medium containing 10% fetal bovine serum (FBS), 2 mM L- Glutamate and 100 units/ml Penicillin/ Streptomycin (medium A) and then, incubated at 37 °C in a 5% CO_2_ humidified atmosphere. After 48 hours, media and unattached cells were removed by washing with *phosphate-buffered saline* (PBS). The adherent cells were maintained in a fresh medium until approximately 80% confluence was reached on day 6 of culture.

**Flow cytometry analysis**

The adherent cells were confirmed to be MSCs by flow cytometry. On day 6, the cells were harvested with trypsin. After centrifuging at 450 g for 5 min, the cells were suspended in DPBS and incubated with following antibodies: FITC (Fluorescein isothiocyanate) conjugated CD45 (BD Biosciences, Cat# 347463, RRID: AB_400306) as leukocyte marker, PE (Phycoerythrin) conjugated CD14 (BD Biosciences, Cat# 347497, RRID: AB_400312) as monocytic marker, FITC conjugated CD44 (BD Biosciences, Cat# 347943, RRID: AB_400360), PE conjugated CD105 (BD Biosciences, Cat# 560839, RRID: AB_2033932) and PE conjugated CD73 (BD Biosciences, Cat # 550257, RRID: AB_393561) for 30 min in the dark. The CD73, CD105 and CD44 served as surface markers of MSCs. Negative control staining was performed by using IgG1- FITC and IgG1-PE isotype controls. Then, the cells were analyzed on Partec *CyFlow Space* cytometer using FloMax software (http://flomax.software.informer. com/ 2.2 /).

**Neuronal differentiation**

On day 6, the medium A was removed and the cells were plated in medium A supplemented with 0.1 mM NEAA, 2% B27 supplement, 1% N2 supplement, 50 ng/ml Noggin, 20 ng/ml EGF and 10 ng/ml recombinant human bFGF (medium B). The medium A was used as the control medium. The growth factors Noggin, EGF and recombinant human bFGF were added to the medium every day. After three days culture in the medium B, the EGF and recombinant human bFGF were removed from the medium (medium C) and the cells were cultured for an additional six days in medium C. The medium was changed every two days.

**RNA extraction and cDNA synthesis**

Total RNA was extracted from untreated and growth factor-treated PBMSCs (day 14) using the total RNA purification kit (Jena Bioscience, Germany) according to the manufacture’s instructions. DNase I treatment of RNA was performed in a final volume of 50 μl containing 40 μl RNA, 5 μl RNase-free DNase I and 5 μl 10x reaction buffer (Fermentas, Thermo Scientific, Waltham, MA, USA). The mixture was incubated for 30 min at 37 °C. Then, the enzyme was inactivated at 65 °C for 10 min. The complete removal of DNA was confirmed by electrophoresis on 1% agarose gel. Finally, the cDNA template was synthesized from extracted RNA using random hexamer primers and dART reverse transcriptase (EURx Ltd, Gdansk, Poland).

**Real time quantitative PCR**

The expression levels of neuronal marker genes were evaluated by quantitative PCR (qPCR) after 14 days of culture. The SYBR Green based qPCR was carried out on Rotor-Gene 6000 Real time PCR system. The qPCR reaction was prepared in a total volume of 25 μl containing 12.5 μl of 2X SYBR Green master mix (Eurex, Poland), 5 μl of the cDNA template, 0.2 μl of each primer (10 pmol/μl) and 7.1 μl of deionized water. A negative control was used by replacing the cDNA template with deionized water. Primer sequences used in this study and their annealing temperature are shown in Table 1.

**Table 1:** Characteristics of qPCR primers pairs used in this study

Gene	Accession number	Primer sequences 5' 3'	Amplicon size (bp)	Annealing temperature (ºC)	Reference
HSP90AB1 (Reference gene)	XM_005249075.1	GGAAGTGCACCATGGAGAGGA	157	55	
		GCGAATCTTGTCCAAGGCATCAG			
β-tubulin III	NM_001197181.1	CTCAGGGGCCTTTGGACATC	160	60	23
		CAGGCAGTCGCAGTTTTCAC			
Nestin	NM_006617.1	ATCGCTCAGGTCCTGGAA	146	60	24
		AAGCTGAGGGAAGTCTTGGA			
NFM	NM_005382.2	GTCAAGATGGCTCTGGATATAGAAATC	104	60	25
		GTCAAGATGGCTCTGGATATAGAAAT			
MAP2	XM_006712533.1	CATGGGTCACAGGGCACCTATTC	233	60	26
		GGTGGAGAAGGAGGCAGATTAGCTG			
NSE	NM_001975.2	GGAGAACAGTGAAGCCTTGG	239	60	27
		GGTCAAATGGGTCCTCAATG			

The PCR amplification consisted of an initial denaturation at 95 ºC for 10 min, followed by 40 cycles of denaturation at 95 ºC for 30 s, annealing at 60 ºC for 30 s and extension at 72 ºC for 30 s. The specificity of PCR products was verified by melting curves and electrophoresis through 3% agarose gel.

The expression level of each gene was calculated as fold change relative to the expression of reference gene (HSP90AB1) using pfaffl method (28). The statistical analysis was performed using Social Science Statistics website (http: //www. socscistatistics.com /tests/studentttest/ Default2. aspx). The *ΔCt* value of treated versus untreated PBMSCs was compared by t-test. Data were represented as *fold change* relative to the cell identifier using GraphPad Prism software (http: //www.graphpad.Com/scientific–software /prism/).

## Results

**Morphological changes of the growth factor-treated PBMSCs**

**Fig. 1:**
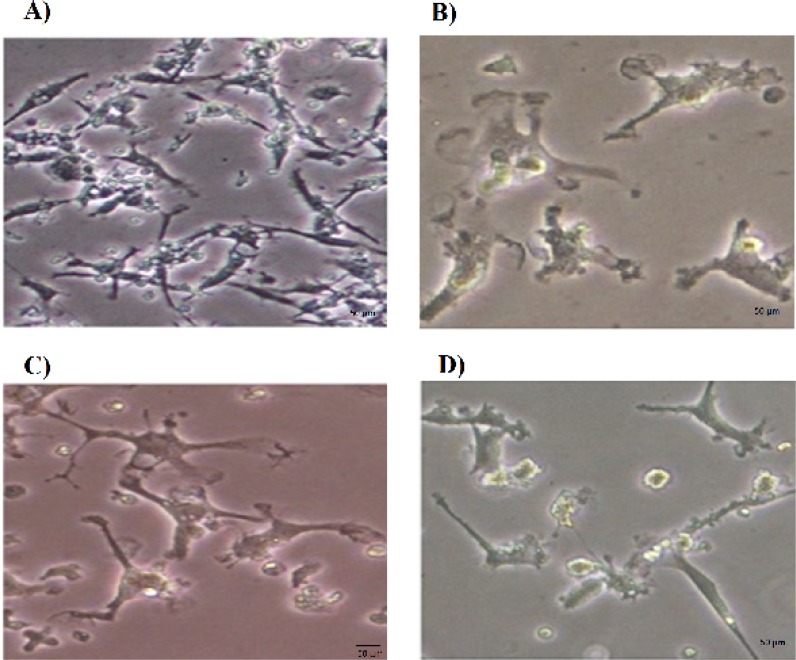
Morphological features of PBMSCs treated with Noggin. A) Prior to treatment, PBMSCs showed fibroblast like shape on day 6. B) PBMSCs after 4 days treatment with Noggin (Day 10). C) PBMSCs after 8 days treatment with Noggin (Day 14). The cells showed the multipolar processes. D) PBMSCs after 8 days treatment with Noggin (Day 14). The cells in this figure displayed synaptic structure

The morphological features of untreated and growth factor-treated PBMSCs were observed under inverted microscope. After being cultured for 6 days, the PBMSCs adhered to the culture surfaces reached 70-80% confluence (Figure 1A). The untreated PBMSCs showed mainly spindle- shaped morphology. These cells had a tendency to become flatter and wider over time. The neurosphere like cells were suspended 2-3 days after culture in the medium induction containing growth factors Noggin, bFGF and EGF. Within 4-5 days after addition of growth factors, some cells began to look like oligodendrocytes or astrocytes. After 8 days treatment with growth factors, the PBMSCs displayed multipolar shapes and bright cell bodies reminiscent of oligodendrocytes morphology (Figure 1C).

**Fig. 2:**
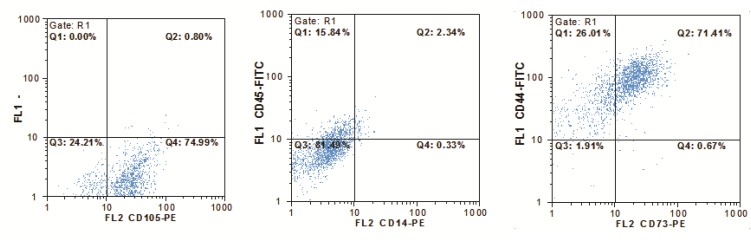
Flow cytometry analysis of adherent cells derived from peripheral blood. The cultured cells were CD45 and CD14 negative. In contrast, presence of MSCs markers (CD73, CD105 and CD44) confirmed that the majority of these cells were MSCs

**Fig. 3:**
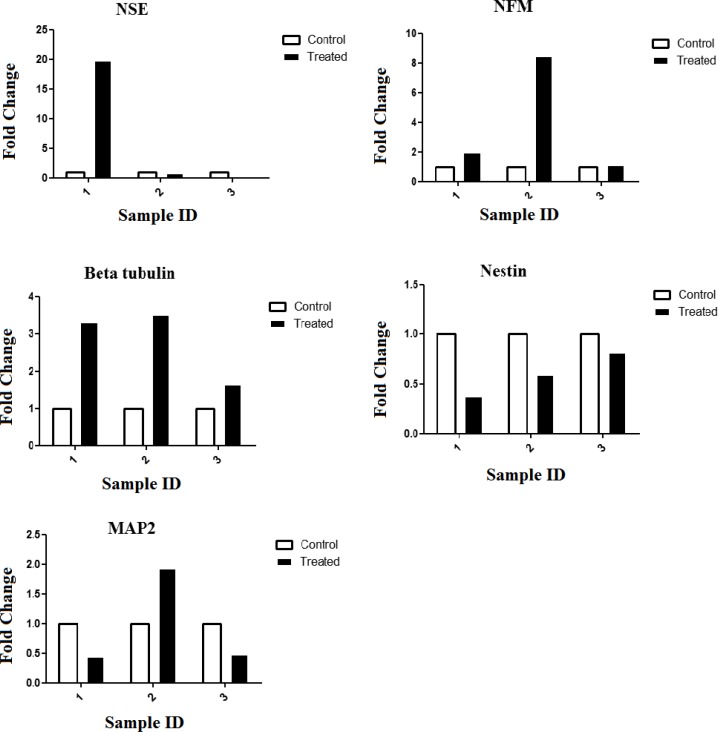
Analysis of neuronal markers expression of the PBMSCs treated with growth factor Noggin. Graphs were generated using GraphPad Prism 6. The results were obtained from three independent experiments

**Expression pattern of surface markers on PBMSCs**

The adherent cells derived from peripheral blood were analyzed by flow cytometry. As expected, these cells were negative for leukocyte marker CD45 as well as monocytic marker CD14. The majority of these cells showed positive signal for mesenchymal cell makers CD105, CD44 and CD73 (Figure 2).

**Expression levels of neural markers in growth factor treated-PBMSCs**

Quantitative analysis with qPCR revealed that the expression of *NFM *and βIII tubulin increased significantly in the growth factor-treated group. Almost three fold increase of βIII tubulin expression was observed upon treatment in the two cultures of PBMSCs. The nestin expression level was markedly reduced in the PBMSCs treated with Noggin. Treatment with Noggin, bFGF and EGF caused an increased expression of *MAP2* and diminished expression of *NSE* in one of the treated-PBMSCs. In contrast, the other culture displayed a reduction of *MAP2* expression and *augmentation* of *NSE* expression after treatment with Noggin. The third culture showed reduced expression of *MAP2* as well as *NSE* in growth factor-treated PBMSCs. The graphs derived from these data are presented in figure 3. The results obtained from statistical analysis indicated that there was *no statistically significant difference between treated and untreated PBMSCs. This is probably due to the low number of experiments.*

## Discussion

Stem cell therapy is a new approach for the treatment of different disorders including neu-rological diseases (29). Different studies have demonstrated that the embryonic stem cells can give rise to neuronal cells (30-31). However, the ethical problems are major concerns in the use of these cells in cell therapy. In some studies, the induced pluripotent stem cells (iPSCs) have been used to establish neuronal stem cells (NSCs) and neuronal lineage cells. The safety problem is an important issue to use of these cells as a therapeutic method (32). MSCs are an alternative source of cells for use in treating patients with neurological disease. It has been demonstrated that these cells have the ability to differentiate into neuron like cells (9). Numerous reports have described different protocols for differentiation of stem cells derived from peripheral blood. The previous studies revealed that PBMCs have ability to differentiate into neural like cells in the presence of different combinations of growth factors (33-34). In this study, a new combination of growth factors including Noggin, bFGF and EGF was used to induce neural differentiation of MSCs derived from peripheral blood (PBMSCs) by inhibition of BMP signaling. To confirm the differentiation of PBMSCs, the expression level of neural cell specific markers were assessed with qPCR.

PB-MSCs showed changes in morphology and expression of neural markers upon treatment with growth factors Noggin, bFGF and EGF. The cells in the present study had morphology different from the neural like cells described in the previous studies. These observations suggested that these cells belonged to different cell types of neural lineage.

Nestin is a marker of NSCs (35) that its expression has also been observed in MSCs (36). It was revealed that the expression of nestin is inversely correlated with cellular differentiation (35). The expression of nestin decreased upon treatment with growth factor Noggin, consistent with differentiation of MSCs. Although the previous studies showed that the expression of nestin needs at least 10 passages of the cultured MSCs in serum free medium (37), but we observed nestin expression in MSCs following culture in medium supplemented with fetal bovine serum for 14 days. These results were consistent with the finding obtained by Foudah et al. (38).

β III tubulin and NFM are known to be the early and late neuronal markers, respectively. These two neural markers are expressed in undifferen-tiated MSCs (39). As observed in Figure 3, Noggin treatment of PBMSCs resulted in increased β III tubulin and *NFM *expression. The results obtained from previous studies suggested that the *in vitro* culture could induce the spontaneous expression of neural markers in MSCs. However, it has remained to be demonstrated (40).

Different isoforms of *MAP2* are expressed in the neural lineage cells (41). The primer pair used in the present study detects *MAP2a*, *MAP2b* and *MAP2c* isoforms. These isoforms of *MAP2* were expressed in different stages of neuronal differen-tiation. MAP2c is an early neuronal marker and its expression decreased in the mature neurons. *MAP2b* was expressed in terminally differentiated neurons as well as during differentiation. *MAP2a* expression was detectable in mature neurons (39). We observed a two-fold increase in the expression of *MAP2 *in one of treated PBMSCs as compared with non-treated PBMSCs. This data along with expression pattern of other markers suggested that PBMSCs differentiated into neuron like cells following treatment with Noggin. In contrast, qPCR analysis showed a decrease of *MAP2* expression in the other two PBMSCs treated with noggin, pro-posing the differentiation of these treated cells into oligodendrocytes. This fact was confirmed by morphology assessment of treated PBMSCs. A previous study on differentiating oligodendrocytes has demonstrated that *MAP2* expression *transiently increased* in preoligodendrocytes. Its expression was decreased at the onset of terminal differentia-tion of oligodendrocytes (42).

Enolase is a key enzyme in the glycolytic pathway which plays an important role in energy production for cells. It has been revealed that γ-enolase (Eno2) was only found in cells of neuronal lineage (43). Our results showed that *NSE* expression level was increased in one of treated cell cultures with Noggin, consistent with their differentiation into neuron like cells. The previous studies indicated that the expression level of *NSE* increased during the oligodendrocyte differentiation and *NSE* expression was repressed in mature oligodendrocytes (44). Furthermore, it has been found that low levels of *NSE* expression are present in astrocytes. Therefore, *NSE* expression data in our study suggested that treatment with Noggin was accompanied by differentiation of PBMSCs into different types of neurons, astrocytes and oligodendrocytes.

In general, our results showed that PBMSCs could express some neural markers including Nestin, βIII tubulin, *NFM*, *MAP2* and *NSE*. Accordingly, PBMSCs are a potential source of cells that can be used to generate neuronal cells. Although different induction protocols were published about differentiation of MSCs into neuron like cells, the introduction of new protocols could improve our understanding from the characteristics of MSCs and the neuron like cells derived from MSCs. Furthermore, the results obtained from this study provide evidence of neuronal differentiation of MSCs upon treatment with Noggin. However, neural marker expression analysis cannot be used as the only proof to demonstrate the neuronal differentiation of MSCs following treatment with Noggin and the complete understanding of these cells needs additional studies from the molecular, biological and physiological aspects.

## Conflict of interest

The authors declared no conflict of interests.

## References

[1] Guo J, Wang H, Hu X (2013). Reprogramming and transdifferen-tiation shift the landscape of regenerative medicine. DNA and cell biology.

[2] Jopling C, Boue S, Izpisua Belmonte JC (2011). Dedifferentiation, transdifferentiation and reprogramming: three routes to regeneration. Nature reviews Molecular cell biology.

[3] Fu YS, Shih YT, Cheng YC (2004). Transformation of human umbilical mesenchymal cells into neurons in vitro. Journal of biomedical science.

[4] Fu YS, Cheng YC, Lin MY (2006). Conversion of human umbilical cord mesenchymal stem cells in Wharton's jelly to dopaminergic neurons in vitro: potential therapeutic application for Parkinsonism. Stem cells.

[5] Toyoda T, Mae S, Tanaka H (2015). Cell aggregation optimizes the differentiation of human ESCs and iPSCs into pancreatic bud-like progenitor cells. Stem cell research.

[6] Gore A, Li Z, Fung HL (2011). Somatic coding mutations in human induced pluripotent stem cells. Nature.

[7] Barry F, Boynton RE, Liu B (2001). Chondrogenic differentiation of mesenchymal stem cells from bone marrow: differentiation-dependent gene expression of matrix components. Experimental cell research.

[8] Jaiswal RK, Jaiswal N, Bruder SP (2000). Adult human mesenchymal stem cell differentiation to the osteogenic or adipogenic lineage is regulated by mitogen-activated protein kinase. The Journal of biological chemistry.

[9] Kim EY, Lee KB, Yu J (2014). Neuronal cell differentiation of mesenchymal stem cells originating from canine amniotic fluid. Human cell.

[10] Oswald J, Boxberger S, Jorgensen B (2004). Mesenchymal stem cells can be differentiated into endothelial cells in vitro. Stem cells.

[11] Chong PP, Selvaratnam L, Abbas AA (2012). Human peripheral blood derived mesenchymal stem cells demonstrate similar characteristics and chondrogenic differentiation potential to bone marrow derived mesenchymal stem cells. Journal of orthopaedic research : official publication of the Orthopaedic Research Society.

[12] Montesinos JJ, Flores-Figueroa E, Castillo-Medina S (2009). Human mesenchymal stromal cells from adult and neonatal sources: comparative analysis of their morphology, immunophenotype, differentiation patterns and neural protein expression. Cytotherapy.

[13] Foudah D, Redondo J, Caldara C (2012). Expression of neural markers by undifferentiated rat mesenchymal stem cells. Journal of biomedicine & biotechnology.

[14] Bae KS, Park JB, Kim HS (2011). Neuron-like differentiation of bone marrow-derived mesenchymal stem cells. Yonsei medical journal.

[15] Guan M, Xu Y, Wang W (2014). Differentiation into neurons of rat bone marrow-derived mesenchymal stem cells. European cytokine network.

[16] Hosseini SM, Talaei-Khozani T, Sani M (2014). Differentiation of human breast-milk stem cells to neural stem cells and neurons. Neurol Res Int.

[17] Nandy SB, Mohanty S, Singh M (2014). Fibroblast Growth Factor-2 alone as an efficient inducer for differentiation of human bone marrow mesenchymal stem cells into dopaminergic neurons. Journal of biomedical science.

[18] Gaulden J, Reiter JF (2008). Neur-ons and neur-offs: regulators of neural induction in vertebrate embryos and embryonic stem cells. Human molecular genetics.

[19] Bais MV, Wigner N, Young M (2009). BMP2 is essential for post natal osteogenesis but not for recruitment of osteogenic stem cells. Bone.

[20] Edgar CM, Chakravarthy V, Barnes G (2007). Autogenous regulation of a network of bone morphogenetic proteins (BMPs) mediates the osteogenic differentiation in murine marrow stromal cells. Bone.

[21] Wang Y, Hong S, Li M (2013). Noggin resistance contributes to the potent osteogenic capability of BMP9 in mesenchymal stem cells. Journal of orthopaedic research : official publication of the Orthopaedic Research Society.

[22] Delaune E, Lemaire P, Kodjabachian L (2005). Neural induction in Xenopus requires early FGF signalling in addition to BMP inhibition. Development.

[23] Jouhilahti EM, Peltonen S, Peltonen J (2008). Class III beta-tubulin is a component of the mitotic spindle in multiple cell types. The journal of histochemistry and cytochemistry : official journal of the Histochemistry Society.

[24] Liu G, Yuan X, Zeng Z (2006). Analysis of gene expression and chemoresistance of CD133+ cancer stem cells in glioblastoma. Molecular cancer.

[25] Mareschi K, Novara M, Rustichelli D (2006). Neural differentiation of human mesenchymal stem cells: Evidence for expression of neural markers and eag K+ channel types. Experimental hematology.

[26] Constantinescu R, Constantinescu AT, Reichmann H Neuronal differentiation and long-term culture of the human neuroblastoma line SH-SY5Y. Journal of neural transmission Supplementum.

[27] Hafizi M, Atashi A, Bakhshandeh B (2013). MicroRNAs as markers for neurally committed CD133+/CD34+ stem cells derived from human umbilical cord blood. Biochemical genetics.

[28] Pfaffl MW (2001). A new mathematical model for relative quantification in real-time RT-PCR. Nucleic acids research.

[29] Lescaudron L, Naveilhan P, Neveu I (2012). The use of stem cells in regenerative medicine for Parkinson's and Huntington's Diseases. Current medicinal chemistry.

[30] Morizane A, Takahashi J, Shinoyama M (2006). Generation of graftable dopaminergic neuron progenitors from mouse ES cells by a combination of coculture and neurosphere methods. Journal of neuroscience research.

[31] Park CH, Minn YK, Lee JY (2005). In vitro and in vivo analyses of human embryonic stem cell-derived dopamine neurons. Journal of neurochemistry.

[32] Velasco I, Salazar P, Giorgetti A (2014). Concise review: Generation of neurons from somatic cells of healthy individuals and neurological patients through induced pluripotency or direct conversion. Stem cells.

[33] Horschitz S, Meyer-Lindenberg A, Schloss P (2010). Generation of neuronal cells from human peripheral blood mononuclear cells. Neuroreport.

[34] Liu Q, Guan L, Huang B (2011). Adult peripheral blood mononuclear cells transdifferentiate in vitro and integrate into the retina in vivo. Cell biology international.

[35] Lendahl U, Zimmerman LB, McKay RD (1990). CNS stem cells express a new class of intermediate filament protein. Cell.

[36] Wong A, Ghassemi E, Yellowley CE (2014). Nestin expression in mesenchymal stromal cells: regulation by hypoxia and osteogenesis. BMC veterinary research.

[37] Wislet-Gendebien S, Hans G, Leprince P (2005). Plasticity of cultured mesenchymal stem cells: switch from nestin-positive to excitable neuron-like phenotype. Stem cells.

[38] Foudah D, Redondo J, Caldara C (2013). Human mesenchymal stem cells express neuronal markers after osteogenic and adipogenic differentiation. Cellular & molecular biology letters.

[39] Chung WJ, Kindler S, Seidenbecher C (1996). MAP2a, an alternatively spliced variant of microtubule-associated protein 2. Journal of neurochemistry.

[40] Croft AP, Przyborski SA (2006). Formation of neurons by non-neural adult stem cells: potential mechanism implicates an artifact of growth in culture. Stem cells.

[41] Shafit-Zagardo B, Kalcheva N (1998). Making sense of the multiple MAP-2 transcripts and their role in the neuron. Molecular neurobiology.

[42] Vouyiouklis DA, Brophy PJ (1995). Microtubule-associated proteins in developing oligodendrocytes: transient expression of a MAP2c isoform in oligodendrocyte precursors. Journal of neuroscience research.

[43] Marangos PJ, Parma AM, Goodwin FK (1978). Functional properties of neuronal and glial isoenzymes of brain enolase. Journal of neurochemistry.

[44] Sensenbrenner M, Lucas M, Deloulme JC (1997). Expression of two neuronal markers, growth-associated protein 43 and neuron-specific enolase, in rat glial cells. Journal of molecular medicine.

